# Non-secretion of AFP and neutrophil lymphocyte ratio as predictors for survival in hepatocellular carcinoma patients treated with sorafenib: a large UK cohort

**DOI:** 10.18632/oncotarget.24769

**Published:** 2018-03-30

**Authors:** Mehran Afshar, Peter Fletcher, Antonio D Bardoli, Yuk Ting Ma, Pankaj Punia

**Affiliations:** ^1^ Oncology, St George's University Hospitals NHS Foundation Trust, London, UK; ^2^ Cancer Research UK Clinical Trials Unit, Birmingham, UK; ^3^ College of Medical and Dental Sciences, University of Birmingham, Birmingham, UK; ^4^ Institute of Immunology and Immunotherapy, University of Birmingham, Birmingham, UK; ^5^ Oncology, Queen Elizabeth Hospital Birmingham, Birmingham, UK

**Keywords:** neutrophil lymphocyte ratio (NLR), alpha-fetoprotein (AFP), hepatocellular carcinoma, sorafenib, survival

## Abstract

**Background:**

Sorafenib is the current standard of care for patients with advanced or metastatic hepatocellular carcinoma. Currently no universally agreed model exists correlating the Neutrophil Lymphocyte ratio (NLR) and non-secretion of AFP with the survival of HCC patients treated with sorafenib.

**Patients and Methods:**

We retrospectively analysed patient records with a confirmed diagnosis of HCC treated with sorafenib between April 2009 and March 2014. Survival analysis was performed using the Kaplan–Meier method and Cox regression.

**Results:**

Patients separated into groups based on NLR (≤3 or >3), or AFP secretion profile (<7 ng/ml or ≥7 ng/ml) derived diverging Kaplan–Meier curves for overall survival (OS). The median OS in those with NLR ≤3.0 was 9.0 months (95% CI: 7.7–11.1 months) and in those with NLR >3.0 it was 6.0 months (95% CI: 4.9–8.2 months) [HR 1.32 (95% CI: 0.96–1.80)]. The median overall survival post sorafenib was higher in the “non-secretor” AFP group. OS for AFP <7 ng/ml was 10.0 months (95% CI: 7.7–19.3 months) compared to AFP ≥7ng/ml: 6.6 months (95% CI: 5.3–8.4 months) [HR 1.64 (95% CI: 1.15–2.33)].

**Conclusion:**

NLR and AFP non - secretion at diagnosis are potential significant prognosticators for overall survival from initiation of sorafenib.

## INTRODUCTION

Hepatocellular carcinoma (HCC) is a highly aggressive malignancy representing the sixth most common cancer and the third leading global cause of cancer related deaths [1, 2]. In the western world the incidence of HCC is still rising [1]. Unfortunately, the majority of patients present at an advanced, non-curative stage.

The Barcelona Clinic Liver Cancer (BCLC) algorithm is an accepted and validated staging system, providing a framework for the management of HCC [3]. Patients with good liver synthetic function and early stage tumour (BCLC 0, A) are eligible for curative treatment options whereas patients with poor liver function and performance status (BCLC D) are advised best supportive care. Patients within BCLC B and C category are eligible for palliative treatment options (loco-regional or systemic). Despite significant improvements in loco-regional and targeted treatment, the long term outcome for patients with advanced HCC remains poor [2].

The ‘SHARP’ and ‘Asia-Pacific’ studies have established sorafenib as the current standard of care for patients with advanced HCC in the first line setting [4, 5]. Sorafenib is an orally active multikinase inhibitor of Raf, Platelet-derived Growth Factor (PDGF) and Vascular Endothelial Growth Factor (VEGF) receptors [6]. A body of evidence exists for the wide heterogeneity of response to sorafenib, but robust prognosticators in this setting are lacking [7–10].

There is increasing evidence that cancer-related inflammation plays an important role in cancer development and progression, through upregulation of cytokines and inflammatory mediators [11]. This is especially the case in HCC which usually arises on a background of chronic hepatitis and cirrhosis. Indeed genomic studies of the adjacent non-neoplastic tissue in HCC following surgical resection have identified a pro-inflammatory gene signature that predicts late recurrence and poor survival [12]. The neutrophil-to-lymphocyte ratio (NLR) is one measure of the systemic inflammatory response with the advantage that it can be readily assessed through a peripheral blood test. An elevated NLR has been associated with a poorer overall survival in many solid tumours [13]. Three recent systematic reviews (the largest involving 20475 patients) have also demonstrated that NLR is a major prognostic factor in HCC; a high baseline NLR was associated with adverse disease-free and/or overall survival in patients with HCC [14–16]. Furthermore, subgroup analyses showed that baseline NLR was prognostic of overall survival for all treatment options in HCC (with the exception of radiofrequency ablation), although the sorafenib group was made up of only two small retrospective studies with 102 and 68 patients respectively [16–18].

Alpha-fetoprotein (AFP) levels have also been investigated as a marker to predict the response of HCC patients after loco-regional treatment or systemic chemotherapy. AFP is a serum glycoprotein that was first identified nearly 50 years ago [19]. It is synthesised in high levels by the foetal yolk sac and foetal liver but its expression is then repressed shortly after birth. Reappearance of AFP in the circulation in adults is associated with chronic hepatitis and liver cirrhosis as well as malignancies such as HCC and gastrointestinal cancers of endodermal origin [20]. AFP is secreted into the blood of approximately 70% of all patients with HCC and higher levels have been found to be associated with a worse prognosis [21–25]. Consequently, AFP has been incorporated into many of the available scoring and staging systems for HCC. However 30–50% of patients with unresectable HCC do not have elevated AFP levels, representing a significant proportion of this group [26, 27]. Patients with low AFP are known to have better prognosis but apart from this, there is a paucity of data regarding prognostic biomarkers in this subgroup of patients.

In this large cohort study of 231 patients at an academic cancer centre in the UK, we aimed to correlate the response to sorafenib with NLR and non-secretion of AFP and assess their validity as prognostic biomarkers for HCC.

## RESULTS

### Baseline characteristics

A total of 231 patients were included in the preliminary notes review. 17 were excluded at first review for unconfirmed diagnosis of HCC or not receiving sorafenib. The remaining 214 selected patients consisted of 172 (79%) males and 45 (21%) females. (Table 1A).

**Table 1A:** Baseline Characteristics NLR groups

		NLR ≤ 3 (*n* = 104)	NLR < 3 (*n* = 110)	All patients (*n* = 214)
**Sex**	Male	82 (79%)	88 (80%)	170 (79%)
	Female	22 (21%)	22 (20%)	44 (21%)
**Age (*n* = 207)**	Median (IQR)	65 (60, 73)	67 (61, 74)	66 (60, 74)
	Range	14 – 86	15 – 82	14 – 86
	≤ 66	56 (55%)	49 (46%)	105 (51%)
	>66	45 (45%)	57 (54%)	102 (49%)
**Child-Pugh**	A	69 (66%)	71 (65%)	140 (65%)
	B	33 (32%)	37 (34%)	70 (33%)
	C	2 (2%)	2 (2%)	4 (2%)
**AFP**	<7 ng/ml	40 (38%)	22 (20%)	62 (29%)
	≥7 ng/ml < 400 ng/mL	40 (38%)	42 (38%)	82 (38%)
	≥ 400 ng/ml	24 (23%)	46 (42%)	70 (33%)
**Portal vein invasion**	Yes	68 (65%)	70 (64%)	138 (64%)
	No	36 (35%)	40 (36%)	76 (36%)
**Aetiology**	ALD	18 (17%)	12 (11%)	30 (14%)
	Hepatitis B	11 (11%)	6 (5%)	17 (8%)
	Hepatitis C	17 (16%)	7 (6%)	24 (11%)
	NASH	8 (8%)	10 (9%)	18 (8%)
	Combination	2 (2%)	10 (9%)	12 (6%)
	Unknown/other	48 (46%)	65 (59%)	113 (53%)
**Previous treatment**	TACE	24 (23%)	32 (29%)	56 (26%)
	RFA	7 (7%)	1 (1%)	8 (4%)
	TACE & RFA	7 (7%)	1 (1%)	8 (4%)
	None	66 (63%)	76 (69%)	142 (66%)

**Table 1B:** Baseline Characteristics AFP secretors groups

		AFP < 7ng/mL (*n* = 62)	AFP ≥ 7 ng/mL (*n* = 152)	All patients (*n* = 214)
**Sex**	Male	56 (90%)	114 (75%)	170 (79%)
	Female	6 (10%)	38 (25%)	45 (21%)
**Age (*n* = 207)**	Median (IQR)	64.5 (55, 71)	67 (61, 75)	66 (60, 74)
	Range	16 – 86	14 – 84	14 – 86
	≤ 66	34 (59%)	71 (48%)	105 (51%)
	> 66	24 (41%)	78 (52%)	102 (49%)
**Child-Pugh**	A	40 (65%)	100 (66%)	140 (65%)
	B	22 (35%)	48 (32%)	70 (33%)
	C	0 (0%)	4 (3%)	4 (2%)
**NLR**	≤ 3.0	40 (65%)	64 (42%)	104 (49%)
	> 3.0	22 (35%)	88 (58%)	110 (51%)
**Portal vein invasion**	Yes	13 (21%)	63 (41%)	76 (36%)
	No	49 (79%)	89 (59%)	138 (64%)
**Aetiology**	ALD	10 (16%)	20 (13%)	30 (14%)
	Hepatitis B	4 (6%)	13 (9%)	17 (8%)
	Hepatitis C	7 (11%)	17 (11%)	24 (11%)
	NASH	6 (10%)	12 (8%)	18 (8%)
	Combination	4 (6%)	8 (5%)	12 (6%)
	Unknown/other	31 (50%)	82 (54%)	113 (53%)
**Previous treatment**	TACE	16 (26%)	40 (26%)	56 (26%)
	RFA	4 (6%)	4 (3%)	8 (4%)
	TACE & RFA	4 (6%)	4 (3%)	8 (4%)
	None	38 (61%)	104 (68%)	142 (66%)

The patients were stratified into two groups according to NLR: Low NLR group <3 (*n* = 104) and high NLR group >3 (*n* = 110). The two groups were generally well balanced for sex, age, Child-Pugh class (CP), portal vein invasion and previous treatments (in categories with more than a few patients), with the low NLR group having a slightly lower age profile. The low NLR group also had a lower AFP profile with more “non-secretors” (AFP <7 ng/ml) and fewer patients with high AFP (>400 ng/ml) than the high NLR group. There were more patients with hepatitis (B and C) and alcoholic liver disease (ALD) in the low NLR group.

We also stratified the patients by AFP secretion status. Non-secretors were defined as having an AFP of <7 ng/ml (*n* = 62) and secretors as having an AFP >7 ng/ml (*n*= 152). We further analysed the baseline characteristics of these AFP secretor groups (Table 1B). Both groups were generally well balanced for age, CP class, aetiological factors for HCC and previous treatments, with the non-secretor group having a slightly lower age profile. There was a marked imbalance between the two groups for sex with a much higher proportion of men in the non-secretor group. There were also more patients with portal vein invasion in the secretor group.

### Overall survival

### NLR

**Figure 1:**
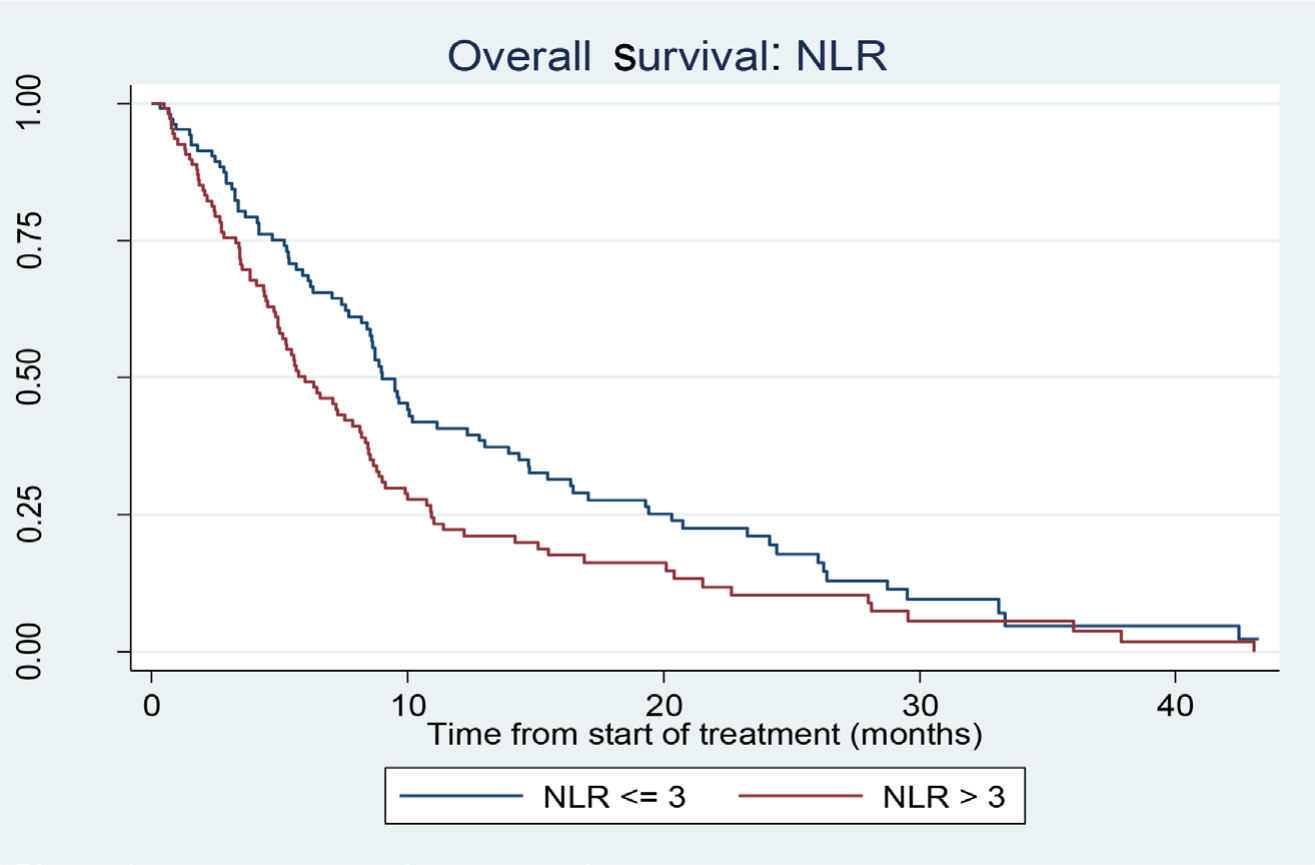
Kaplan Meier curve showing survival in patients with NLR ≤ 3 versus NLR > 3

The median overall survival in those with NLR ≤3.0 was 9.0 months (95% CI: 7.7–11.1 months) and in those with NLR >3.0 it was 6.0 months (95% CI: 4.9–8.2 months). (Figure 1). The hazard ratio of NLR < 3 to NLR ≤3 indicate that higher NLR is associated with shorter survival times (HR1.45 (95% CI: 1.07, 1.95)). When adjusted for CP class, portal vein invasion and AFP the trend was still apparent (HR1.32 (95% CI: 0.96, 1.80)). This statistical significance was lost when the NLR ratio was increased to 4 or 5 but HR trend was maintained. Loss of statistical significance in our cohort may be related to small sample size (Table 2).

**Table 2:** Overall Survival stratified by NLR

	Unadjusted (*n* = 214)		Adjusted^1^ (*n* = 214)
**NLR**	**Number (%) in higher group**	**HR (95% CI)**	**HR (95% CI) ^3^**
**3**	110 (51%)	1.45 (1.07, 1.95)	1.32 (0.96, 1.80)
**4**	62 (29%)	1.22 (0.88, 1.69)	1.27 (0.91, 1.77)
**5**	42 (20%)	1.47 (1.01, 2.13)	1.39 (0.95, 2.04)

Adjusted for Child-Pugh class, portal vein invasion and ln(AFP).

A log-rank test comparing NLR < 3 with NLR ≥ 3 found a statistically significant difference between the survival outcomes for the two groups: (*p* = 0.015). A log-rank test stratified by CP class, portal vein invasion and AFP level (<7 ng/ml) was marginally significant (*p* = 0.055). We used backwards elimination method for significant variables.

### AFP

**Figure 2:**
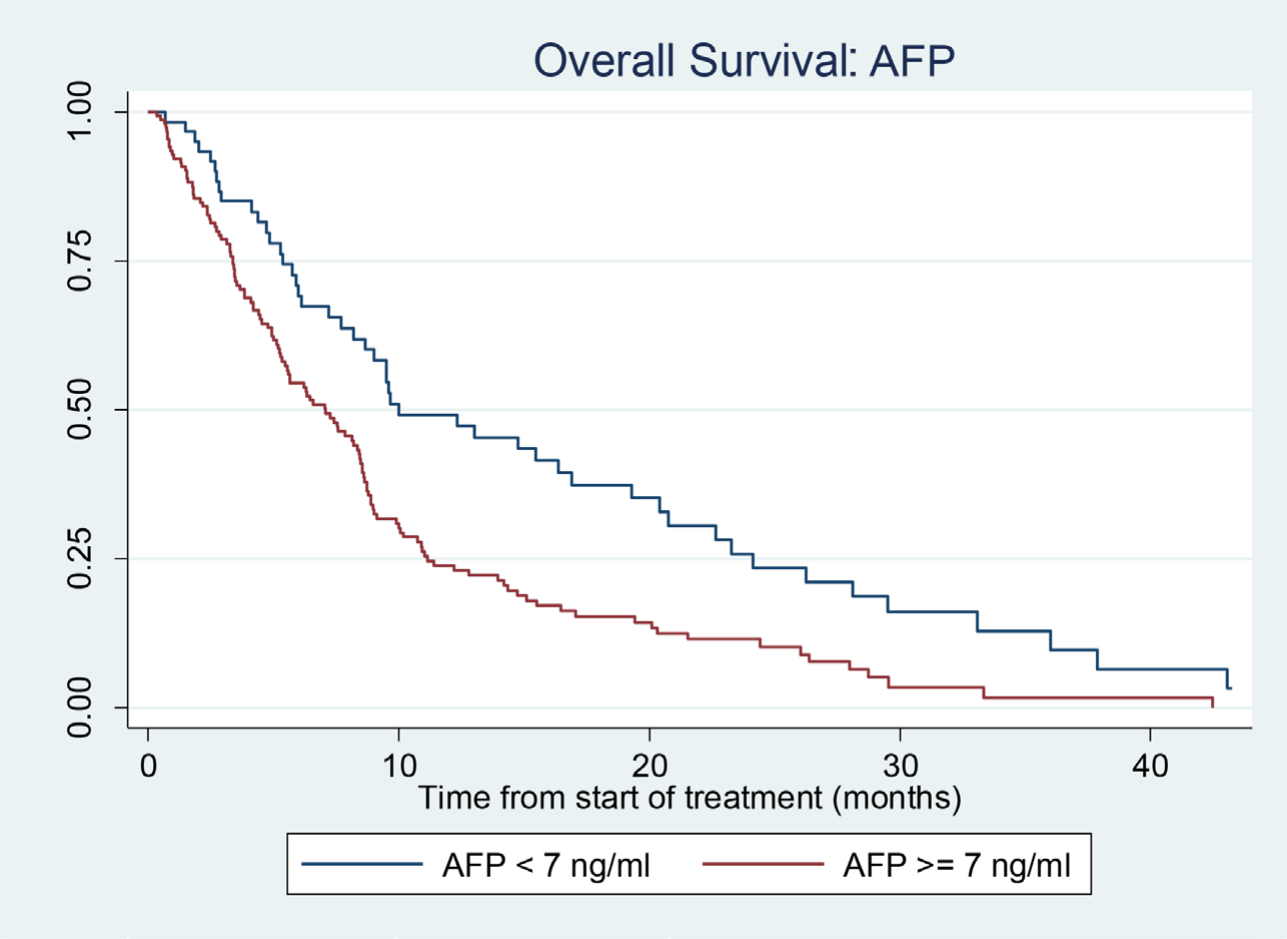
Kaplan Meier curve showing survival in patients with AFP < 7 and AFP ≥ 7

The median overall survival post sorafenib was higher in the “non-secretor” AFP groups. OS for AFP <7 ng/ml was 10.0 months (95% CI: 7.7–19.3 months) compared to AFP ≥ 7 ng/ml: 6.6 months (95% CI: 5.3–8.4 months). (Figure 2). The non-secretors of AFP showed an unadjusted HR of 1.87 (95% CI 1.3–2.6) and an adjusted HR of 1.64 (95% CI: 1.2–2.3) for CP class, portal vein invasion, previous treatment.

A log-rank test comparing AFP < 7 ng/ml with AFP = >7 ng/ml found a highly statistically significant difference between the survival outcomes for the two groups: (*p* = 0.0003). A log-rank test stratified by CP class, portal vein invasion and previous localised treatments (RFA/TACE) was also statistically significant (*p* = 0.0080).

## DISCUSSION

Sorafenib was established as the first treatment to improve overall survival in patients with advanced HCC in 2008 [4, 5], yet to date, there remains no validated predictive or prognostic biomarker for its use. We show that NLR and non-secretion of AFP have potential to be prognostic biomarkers in this setting with the additional advantages of being readily available and inexpensive.

In this large single-centre cohort of consecutive patients treated with sorafenib, we observed that patients with an elevated pre-treatment NLR (>3) have a significantly worse overall survival following treatment with sorafenib compared with patients with a NLR ≤3. Our results are consistent with the emerging data in this area and adds to the evidence base; since we embarked on this study there are now three small retrospective studies and one large observational study that have all demonstrated that elevated pre-treatment NLR is associated with poorer TTP and/or overall survival following sorafenib treatment, after adjusting for clinical covariates [17, 18, 28, 29]. One major limitation of the existing studies is that all of the studies (including ours) have been observational and mostly retrospective in nature, and thus the prognostic ability of NLR still requires prospective evaluation.

During the writing of this manuscript, exploratory analyses from two prospective studies performed in patients with advanced HCC have been published: Personeni *et al.* analysed the prognostic value of NLR in patients treated in the ARQ 197–215 study, a randomised placebo-controlled study testing the MET inhibitor tivantinib in the second line setting for patients with advanced HCC [30]. Multivariate analysis revealed that baseline NLR was an independent prognostic biomarker in this setting; NLR >3 was associated with poorer survival (HR1.65; 95% CI 1.05; 2.59; *p* = 0.03) [30]. Bruix *et al.* conducted a pooled exploratory analysis from the 2 landmark placebo-controlled phase 3 studies (SHARP and Asia-Pacific) in an attempt to identify prognostic factors for OS and predictive factors of sorafenib benefit [31]. A high NLR (≤ vs >median [3.1]) was a strong and independent prognostic factor for overall survival. Furthermore, the OS benefit of sorafenib was consistently observed in all subgroups but patients with a low NLR derived a greater OS benefit from sorafenib treatment (HR, 0.59 vs 0.84) [31]. Thus in advanced HCC the available literature now robustly supports NLR as an independent prognostic factor for survival in this group, independent of treatment allocation, in both the first line and second line setting.

Another important finding of our study is that patients who do not secrete AFP have a significantly improved OS. Although low AFP is known to be associated with better outcomes in HCC following surgical resection and liver transplantation, to our knowledge, this is the first time this has been reported for patients undergoing sorafenib treatment.

There is a paucity of research on patients with low AFP and HCC and in particular potential prognostic factors in this subgroup. In our study, we found no evidence that NLR ratio <>3 was associated with survival in the AFP non-secretor subgroup, however the numbers of patients contributing to this analysis was only small. However, a unique finding from our study is the association of low AFP with low NLR, with a much higher proportion of patients with low NLR in the AFP non-secretor group compared to the AFP secretors. This has not previously been reported. Whilst prospective validation of this finding is needed, this association may in part explain the better prognosis of patients with HCC who do not secrete AFP. Recent *in vitro* studies have demonstrated that tumour-derived AFP can directly impair natural killer (NK) cell activation and viability [32], indirectly impair NK cell activity through suppression of dendritic cell function [33], and induce aberrant dendritic cell differentiation with consequential reduction in the secretion of inflammatory cytokines and chemokines and limited T cell activation [34, 35]. Thus shifting the balance towards a high NLR in the presence of AFP.

There has only been one other study which has looked at prognostic biomarkers in the subset of patients with low AFP. Carr *et al.* demonstrated that serum gamma glutamyl transpeptidase (GGGT) levels correlated with survival in patients with unresectable HCC and low AFP [27]. As GGGT is also a readily available test, we recommend prospective evaluation of the combination of NLR and GGGT in patients with HCC and non-secretion of AFP.

Other prognostic biomarkers associated with sorafenib have also been investigated. In the the SHARP trial, Llovet *et al.* analysedplasma biomarkers- Ang2, bFGF, VEGF, sVEGFR-2,sVEGFR-3, HGF, s-c-KIT, IGF-2 and Ras after 12 weeks of treatment with sorafenib [36]. This revealed that only Ang2 and VEGF were independent predictors of survival but none of the biomarkers were predictive of response to treatment [36]. Furthermore Masuda *et al*. recently reported that expression of high mobility group box 1 (HMGB1) at 4 weeks was an independent predictor of poor overall survival with sorafenib treatment (*P* = 0.001) [37]. However the cost-effectiveness and availability of these markers in routine clinical practise are significant limitations. The advantage of NLR and AFP is that they are both inexpensive routine blood tests which are frequently used when planning HCC treatment.

In summary, we have shown that NLR and non-secretion of AFP both have potential as prognostic biomarkers in patients with advanced HCC treated with sorafenib. Further prospective validation of our findings is required, in particular the association of low AFP and low NLR, but if these findings are validated then these may provide clinicians with inexpensive and routinely available biomarkers to better help select patients for future treatment and clinical trials.

## PATIENTS AND METHODS

All patients with a confirmed diagnosis of HCC, either histologically or radiologically as per internationally accepted American Association for Study of Liver Diseases (AASLD) criteria, who commenced treatment with sorafenib at our centre between April 2009 and March 2014 were included in this analysis.

Electronic patient records were reviewed and variables including demographics, haematological and biochemical laboratory results, radiological data and survival outcomes were collected. Survival analysis was performed using the Kaplan-Meier method, Cox regression and log-rank tests. All analyses were performed in Stata 14.

## Abbreviations

HCC: Hepatocellular carcinoma
NLR: Neutrophil Lymphocyte ratio
AFP: Alpha-fetoprotein
OS: overall survival
CI: confidence intervals
BCLC: Barcelona Clinic Liver Cancer Algorithm
PDGF: platelet derived growth factor
VEGF: vascular endothelial growth factor
AASLD: American Association for the study of Liver Disease
CP: Child Pugh
HR: hazard ratio
ALD: alcoholic liver disease
NASH: non-alcoholic steatohepatitis
TACE: transarterial chemoembolization
RFA: radiofrequency ablation
NK cells: natural killer cells
Ang-2: angiopoietin-2
BFGF: basic fibroblast growth factor
VEGFR: vascular endothelial growth factor receptor
HGF: hepatocyte growth factor
S-C-KIT: soluble receptor c-KIT
IGF-1: insulin-like growth factor
HMGB1: high mobility group box 1
